# Effects of calcium–magnesium carbonate and calcium–magnesium hydroxide as supplemental sources of magnesium on microbial fermentation in a dual-flow continuous culture

**DOI:** 10.1093/tas/txaa229

**Published:** 2020-12-22

**Authors:** J A Arce-Cordero, H F Monteiro, V L N Brandao, X Dai, S L Bennett, A P Faciola

**Affiliations:** Department of Animal Sciences, University of Florida, Gainesville, FL

**Keywords:** alkalinizer, buffer, mineral, ruminal acidosis

## Abstract

Supplemental sources of Mg can also aid in ruminal pH regulation due to their alkaline properties. Magnesium oxide (MgO) is the most common source of Mg for ruminants and can help controlling ruminal pH; however, the alkaline potential of other sources of Mg has not been evaluated. We aimed to evaluate the inclusion of calcium–magnesium carbonate (CaMg(CO_3_)_2_) and calcium–magnesium hydroxide (CaMg(OH)_4_) alone or in combination as supplemental sources of Mg in corn silage-based diets and its impact on ruminal microbial fermentation. We hypothesized that inclusion of CaMg(OH)_4_ would allow for ruminal fermentation conditions resulting in a greater pH compared to the inclusion of CaMg(CO_3_)_2_. Four treatments were defined by the supplemental source of Mg in the diet: 1) Control (100% MgO, plus sodium sesquicarbonate as a buffer); 2) CO_3_ [100% CaMg(CO_3_)_2_]; 3) OH [100% CaMg(OH)_4_]; and 4) CO_3_/OH [50% Mg from CaMg(CO_3_)_2_, 50% Mg from CaMg(OH)_4_]. Nutrient concentration was held constant across treatments (16% CP, 30% NDF, 1.66 Mcal NEl/kg, 0.67% Ca, and 0.21% Mg). Four fermenters were used in a 4 × 4 Latin square design with four periods of 10 d each. Samples were collected for analyses of nutrient digestibility, soluble Mg, VFA, and NH_3_, while pH was measured at 0, 1, 2, 4, 6, 8, and 10 h post morning feeding to estimate % time when pH was below 6 (pH-B6) and area under the pH curve for pH below 6.0 (pH-AUC). Bacteria pellets were harvested for ^15^N analysis and estimates of N metabolism. Treatment effects were analyzed with the mixed procedure of SAS, while effects of using either CaMg(CO_3_)_2_ or CaMg(OH)_4_ as Mg source in comparison to Control treatment were evaluated by orthogonal contrasts. Similar pH-related variables were observed for Control, OH, and CO_3_/OH treatments, which had smaller pH-AUC and pH-B6 than CO_3_ (*P* ≤ 0.01). Butyrate molar proportion was greater in Control and CO_3_/OH than in CO_3_ and OH (*P* = 0.04). Orthogonal contrasts showed lower flow of bacterial N (*P* = 0.04), lower butyrate molar proportion (*P* = 0.08) and greater pH-AUC (*P* = 0.05) for diets with CaMg(CO_3_)_2_ in comparison with the Control. Concentration of soluble Mg in ruminal fluid (*P* = 0.73) and nutrient digestibility (*P ≥* 0.52) were similar across treatments. Under the conditions of this experiment, using CaMg(OH)_4_ alone or combined with CaMg(CO_3_)_2_ allowed for a less acidic ruminal fermentation pattern than a diet with only CaMg(CO_3_)_2_.

## Introduction

Animals require Mg as an enzymatic cofactor involved in energy metabolism, DNA synthesis and cell growth; and also for adequate functioning of muscle cells, signal transmission of nerves and structural component of bones (Schonewille and Beynen, 2005).

In ruminants, maintaining an adequate status of Mg is greatly dependent on ruminal absorption of this nutrient, which relies on the solubility of Mg sources (NRC, 2001; Goff, 2018). In this regard, magnesium oxide (MgO) is the most common supplemental source of Mg due to its ruminal solubility and absorption (NRC, 2001).

Because of their alkaline properties, supplemental sources of Mg can aid in ruminal pH control. Supplementation with MgO has shown effective to prevent a decline in ruminal pH, fiber digestibility, and milk fat production; accounting for similar effects than those observed with sodium bicarbonate buffer (Erdman et al., 1982; Bach et al., 2018). However, much less is known about other sources of Mg that could potentially provide an alkalinizing effect in the rumen.

Calcium magnesium carbonate (CaMg(CO_3_)_2_) has been fed as a source of Mg to ruminants, with an estimated bioavailability ranging from lower (Ammerman et al., 1972; Rahnema and Fontenot, 1983) to similar (Leno et al., 2017) to that one observed for MgO; however, its effects on ruminal fermentation are still not clear. Furthermore, to the best of our knowledge calcium magnesium hydroxide (CaMg(OH)_4_), resulting from the hydration of calcium magnesium oxide, has not been evaluated yet as a source of Mg for ruminants. However, due to its alkaline nature, it may influence ruminal pH when used as a supplemental source of Mg.

According to their chemical composition, theoretical neutralizing capacities of CaMg(CO_3_)_2_ and CaMg(OH)_4_ correspond to 21.7 and 60.4 mEq/g, respectively; indicating a greater alkalinizing potential for CaMg(OH)_4_. Based on these theoretical values, we performed a preliminary test of reactivity in acetic acid according to Goff (2018). In agreement with theoretical neutralizing capacity, estimated reactivity of CaMg(OH)_4_ was approximately three times greater than CaMg(CO_3_)_2_ reactivity. Therefore, because of its chemical properties, when included in diets for ruminants, CaMg(OH)_4_ may provide a greater alkaline effect than CaMg(CO_3_)_2_.

In this experiment, we evaluated supplementation of different inorganic sources of Mg at the levels recommended by NRC (2001) for lactating dairy cows’ diets. Our objective was to evaluate CaMg(CO_3_)_2_ and CaMg(OH)_4_ alone or in combination as supplemental sources of Mg and their impact on microbial ruminal fermentation in a continuous culture system. We hypothesized that inclusion of CaMg(OH)_4_ would allow for ruminal fermentation conditions resulting in a greater pH compared to the inclusion of CaMg(CO_3_)_2_.

## MATERIALS AND METHODS

### Experimental Design and Diets

The University of Florida Institutional Animal Use and Care Committee approved all the procedures for animal care and handling required for this experiment. Four fermenters of a dual-flow continuous culture system, with an average volumetric capacity of 1.82 L each, were used for this experiment in a 4 × 4 Latin square design with treatments defined by the supplemental source of Mg in the diet, as follows: 1) Control: 100% supplemental Mg from MgO, plus 0.6% sodium sesquicarbonate; as a positive control treatment; 2) CO_3_: 100% supplemental Mg from CaMg(CO_3_)_2_; 3) OH: 100% supplemental Mg from CaMg(OH)_4_; and 4) CO_3_/OH: 50% supplemental Mg from CaMg(CO_3_)_2_ and 50% from CaMg(OH)_4_. As shown in Table 1, experimental diets were formulated to provide the same concentration of nutrients regardless of treatment and according to the NRC (2001) recommendations for lactating Holstein cows with 680 kg body weight, and milk production of 45 kg/d with 3.5% fat, 3.0% protein, and 4.8% lactose.

**Table 1.** Ingredient and chemical composition of experimental diets

Item	Treatment^*a*^			
	Control	CO_3_	OH	CO_3_/OH
Item, %DM				
Corn silage	34.92	35.10	35.14	35.12
Ground corn	33.31	33.48	33.51	33.49
Soybean meal 48% CP	18.61	18.71	18.73	18.72
Grass hay	9.80	9.85	9.86	9.85
CaCO_3_	1.34	1.18	1.18	1.18
White salt	0.49	0.49	0.49	0.49
Trace mineral premix	0.49	0.49	0.49	0.49
Calcium phosphate	0.34	0.34	0.34	0.34
Sodium sesquicarbonate	0.60	–	–	–
MgO	0.10	–	–	–
CaMg(CO_3_)_2_	–	0.35	--	0.18
CaMg(OH)_4_	–	–	0.26	0.13
Chemical composition, %DM^*b*^				
CP	16.1	16.2	16.2	16.2
EE	3.1	3.1	3.1	3.1
NDF	30.3	30.5	30.5	30.5
Starch	33.1	33.3	33.3	33.3
NEl, Mcal/kg	1.66	1.66	1.67	1.66
K	1.16	1.17	1.17	1.17
Ca	0.66	0.68	0.67	0.67
P	0.35	0.35	0.35	0.35
Mg	0.21	0.21	0.21	0.21

^*a*^Experimental treatments based on supplemental source of Mg: “Control” = 100% as MgO + sodium sesquicarbonate added as a buffer; “CO_3_” = 100% as CaMg(CO_3_)_2_; “OH” = 100% as CaMg(OH)_4_; “CO_3_/OH” = 50% as CaMg(CO_3_)_2_/50% as CaMg(OH)_4_.

^*b*^Expressed as a percent of DM unless otherwise stated.

Nutrient composition of feeds was determined in samples ground through a 1 mm screen in a Wiley mill (model no. 2; Arthur H. Thomas Co., Philadelphia, PA). Major feed ingredients for experimental diets fed to the fermenters (corn silage, corn grain, soybean meal, and grass hay) were ground through a 2 mm screen using the same type of mill described above. Before grinding, corn silage was dried for 72 h at 60 °C in a forced-air oven (Heratherm, Thermo Scientific, Waltham, MA) until %DM was approximately 90% allowing for proper grinding and storage.

The sources of Mg evaluated in this experiment contained 48.2% Mg (MgO), 13.3% Mg and 20.6% Ca (CaMg(CO_3_)_2_), and 18.5% Mg and 24.2% Ca (CaMg(OH)_4_), on a dry basis. Reactivity of Mg sources was evaluated according to Goff (2018) as an indicator of alkalinizing potential and solubility. Briefly, for each source, 3.0 g were weighed into a 225 mL plastic container, then 40 mL of a commercial 5% dilution of acetic acid were slowly added and shaken by hand for 15 s. Then, the mix was let sit for 15 min and shaken for 15 s again. Finally, 30 min after acetic acid was added, the pH of the mix was measured with a portable pH meter probe (Thermo Scientific Orion Star A121) and used as an indicator of reactivity. Each source was analyzed by triplicate and the average final pH was 3.52, 3.34, and 12.22 for MgO, CaMg(CO_3_)_2_, and CaMg(OH)_4_, respectively.

### Dual-Flow Continuous Culture System Operation

A dual-flow continuous culture system originally described by Hoover et al. (1976) and evaluated by Brandao and Faciola (2019) and Brandao et al. (2020) was used for this experiment. Ruminal fermentation is simulated in this system through continuous agitation (100 rpm), infusion of N_2_ gas to displace oxygen, constant temperature (39 °C), and infusion of artificial saliva (Weller and Pilgrim, 1974) with 0.40 g/L of urea, at 3.05 mL/min to individually regulate passage rates of liquid (11%/h) and solid (5.5%/h) effluents of digesta.

This experiment consisted of four fermentation periods of 10 d each (40 d of fermentation total). Fermenters were inoculated on day 1 of each fermentation period with fresh ruminal contents collected from two cannulated Holstein cows in midlactation (108 ± 9 DIM on average) fed twice a day a total mixed ration with 38% corn silage, 19% ground corn, 13% soybean meal, 11% cottonseed, 9% citrus pulp, 8.5% mineral premix, and 1.5% palmitic acid supplement (on a DM basis) from 3 wk before the start and until completion of the experiment. Approximately 1 h after morning feeding, ruminal contents were manually collected from each cow and strained through two layers of cheesecloth, transferred into prewarmed thermos jars, and immediately transported to the lab. Each fermenter was pre-warmed and under the continuous flush of N_2_ gas at the moment of inoculation when it was filled with a 50:50 mix (v/v) of ruminal contents from both cows.

Each fermenter was provided with its corresponding experimental diet (106 g DM/d) distributed into two identical portions of 53 g DM at 0800 and 1800 h. On d 5 of each period, a pulse dose of 0.1733 g (^15^NH_4_)_2_SO_4_ 10.2% atom excess (Sigma Aldrich Co.) was provided to each fermenter to create a steady-state of ^15^N, and then (^15^NH_4_)_2_SO_4_ was continuously added as a marker in artificial saliva at a rate of 0.077g/L until the end of that fermentation period. From d 8 to d 10 of fermentation, containers of solid and liquid digesta effluent were kept in an ice-cold water bath and digesta temperature maintained below 2 °C to prevent any further microbial fermentation of nutrients happening outside of the fermenters. On day 10 upon completion of each period, all fermenters were put apart and cleaned, disposable components of the system were replaced, and fermenters were reassembled, and rerandomized into experimental treatments for the following period.

### Collection of Data and Samples

Data of pH and daily effluents of digesta, and samples for analyses of nutrient digestibility, VFA, ammonia N (NH_3_-N), the concentration of soluble Mg, and ^15^N concentration (N metabolism), as shown below.

#### Nutrient digestibility.

Effluent samples (liquid and solid) were collected on days 8, 9, and 10 of each period for estimates of ruminal digestibility of nutrients. Liquid and solid digesta effluents of the same fermenter (representing 24 h of fermentation) were weighed, combined and stored at −20 °C. Samples of digesta from the same fermenter and period were pooled across the three collection days, then a 200 mL composite sample per fermenter per period was freeze-dried, ground with a mortar and pestle, and stored in a plastic container for further analyses to determine ruminal digestibility of OM, CP, and NDF in duplicates.

#### Ruminal pH.

On days 8, 9, and 10 of each period, pH was measured in each fermenter at 0, 1, 2, 4, 6, 8, and 10 h after morning feed provision. These data were used for estimation of daily average pH, time below pH 6.0 (pH-B6) expressed as the percentage of the time during the measurement period that pH was below the threshold of 6.0, and area under the pH curve (pH-AUC) expressed as the area under the curve when the pH was below 6.0 and estimated with the trapezoidal rule (Cardoso et al., 2011). Equations used for pH-B6 and pH-AUC calculations are shown below:

$$\begin{array}{ll} pH\text{-}B6\;(\;\%\;)= & \;\;100[time\;below\;threshold\;\\ & \left(h/d\right))/length\;of\;daily\;pH\\ & \;monitoring\;time\;\left(h\right)], \end{array}$$

$$\begin{array}{l} pH\text{-}AUC(\;pH\cdot h)=\sum \left[(pH_{a}+pH_{b})(t_{b}-t_{a})/2\right], \end{array}$$

where pH_a_ and pH_b_ represent the measured pH values of each interval between timepoints at times t_a_ and t_b_, respectively.

#### Daily concentrations of Mg, VFA, and NH_3_-N.

 On days 8, 9, and 10 of each period two 10 mL samples of mixed digesta effluent (solid and liquid) were collected from each fermenter, strained through four layers of cheesecloth, and collected into two separate 15 mL centrifuge tubes. The sample for Mg solubility analysis was collected in an empty 15 mL metal-free centrifuge tube, while the sample for VFA and NH_3_-N was collected in a 15 mL centrifuge tube acidified with 100 µL of 50% H_2_SO_4_, and both samples were stored at −20°C immediately after collection.

#### Nitrogen metabolism.

On day 5 of each period before ^15^N enriched saliva started being infused in the system, a 200 mL sample of ^15^N enriched saliva and a 200 mL sample of digesta effluent (liquid and solid) from each fermenter were collected to measure the concentration of ^15^N in enriched saliva and ^15^N background concentration in digesta, respectively. On days 8, 9, and 10, a 200 mL sample of effluent (liquid and solid) was collected from each fermenter for measurement of ^15^N concentration in digesta effluent. Also, on day 10 of each period, the whole content of each fermenter was processed for isolation of bacteria according to Krizsan et al. (2010). Briefly, fermenter contents were blended for 30 s, filtered through four layers of cheesecloth, and washed with 400 mL of 0.9% saline solution. The filtrate was centrifuged (Sorvall RC-5B Refrigerated Superspeed Centrifuge, DuPont Instruments) at 1,000 × *g* for 10 min and the resulting supernatant was centrifuged at 11,250 × *g* for 20 min to obtain the pellet of bacteria that was resuspended in 200 mL of McDougall’s solution, and centrifuged at 16,250 × *g* for 20 min. Finally, the bacteria pellet was transferred with distilled water into a plastic container for storage.

Immediately after collection, samples of ^15^N enriched saliva, ^15^N background, digesta effluent, and bacteria, were stored at −20 °C until freeze-dried. Dried samples were first ground with a mortar and pestle and then ball-milled at 25 Hz for 9 min using a Mixer Mill MM400 (Retsch, Newton, PA) for analysis of ^15^N.

### Laboratory Analyses

#### Chemical composition**.**

Samples of dietary feed ingredients (corn silage, grass hay, corn grain, and soybean meal) and freeze-dried digesta samples were analyzed for DM (AOAC, 1990; method 930.15), ash (AOAC, 1990; method 942.05), NDF (Van Soest et al., 1991) adapted for Ankom^200^ Fiber Analyzer (Ankom Technology, Macedon, NY) with heat-stable α-amylase and sodium sulfite, and total N (AOAC, 2000; method 990.03) by rapid combustion with a micro elemental N analyzer (Vario Micro Cube, Elementar, Hanau, Germany). Additionally, feed ingredients used for experimental diets were analyzed for concentration of total starch by enzymatic hydrolysis (AOAC, 2000; method), and Ca, P, and Mg by inductively coupled plasma mass spectrometry (AOAC, 2000; method 985.01).

#### Volatile fatty acids.

Concentrations of acetate, propionate, butyrate, isobutyrate, isovalerate, valerate, and caproate were analyzed by gas chromatography (Agilent 7820A GC, Agilent Technologies, Palo Alto, CA) with a flame ionization detector and a capillary column (CP-WAX 58 FFAP 25 m 0.53 mm, Varian CP7767, Varian Analytical Instruments, Walnut Creek, CA) that was maintained at 110 °C, with injector temperature at 200 °C and detector at 220 °C. Samples were thawed at room temperature and processed according to Ruiz-Moreno et al. (2015) by centrifuging at 10,000 × *g* for 15 min, mixing the supernatant with a solution of crotonic acid and metaphosphoric acid to freeze overnight, and then centrifuged again at 10,000 × *g* for 15 min. The supernatant was mixed with ethyl acetate, vortexed and allowed to settle to finally transfer the top layer to a chromatography injection vial for analysis.

#### Ammonia nitrogen.

Analysis of NH_3_-N in ruminal fluid samples was done according to Broderick and Kang (1980). Samples were thawed at room temperature, centrifuged at 10,000 × *g* for 15 min, and the supernatant used for analysis by the phenol–hypochlorite method in a 96-well flat-bottom plate. Absorbance was measured at 620 nm using a spectrophotometer (SpectraMax Plus 384 Microplate Reader).

#### Soluble Mg.

Analysis of soluble Mg in ruminal fluid samples was performed according to Jittackhot et al. (2004). Briefly, samples were centrifuged at 18 °C at 2,700 × *g* for 15 min, then the supernatant was centrifuged 20 °C at 30,000 × *g* for 30 min, and an aliquot was collected for Mg analysis by inductively coupled plasma mass spectrometry (AOAC, 2000; method 985.01).

#### Percent atom ^15^N analysis.

Based on expected ^15^N concentrations 1, 2, or 4 mg of ball-milled sample (for samples of bacteria, digesta effluent, or background, respectively) were weighed into an 8 × 5 mm pressed standard-weight tin capsule (Elemental microanalysis, Devon, UK) using a Mettler Toledo Excellence Plus XP Micro Balance (Mettler-Toledo GmbH, Laboratory & Weighing Technologies, CH-8606 Greifensee, Switzerland), then 35 µL of K_2_CO_3_ solution (10 g/L) were added to each sample and left overnight in a forced-air oven at 40 °C for complete evaporation of NH_3_-N. Finally, % atom ^15^N in samples was determined by isotope ratio mass spectrometry (IsoPrime 100, IsoPrime, Manchester, UK) and expressed as the fractional abundance of isotopic fractions (^15^N/^14^N) multiplied by 100. Furthermore, these samples were analyzed for DM (AOAC, 1990; method 930.15), ash (AOAC, 1990; method 942.05), and concentration of total N (AOAC, 2000; method 990.03) by rapid combustion with a micro elemental N analyzer (Vario Micro Cube, Elementar, Hanau, Germany).

### Calculations for N Metabolism and Digestibility of Nutrients

Total N in digesta effluent was partitioned into three main fractions: NH_3_-N flow, dietary N flow (undigested N), and bacterial N flow. The two latter fractions combined are also known as nonammonia N (NAN).

Flows of NH_3_-N and NAN were determined according to Bach and Stern (1999), while the flow of bacterial N was determined according to Calsamiglia et al. (1996) using the following equations:

$$\begin{array}{l} \begin{array}{ll} NH_{3}\text{-}N\;flow\;\left(g/d\right)=\;\; & NH_{3}\text{-}N\;concentration\;in\;\\ & effluent\;\left(mg/dL\right)\\ & \times (mL\;of\;total\;effluent\;flow/100), \end{array} \end{array}$$

$$\begin{array}{ll} NAN\;flow\;\left(g/d\right)= & \;\;g\;of\;total\;N\;in\;effluent\\ & \;-g\;of\;effluent\;NH_{3}\text{-}N, \end{array}$$

$$\begin{array}{ll} & Bacterial\;N\;flow\;\left(g/d\right)\\ & =\frac{\begin{array}{lll} & ( & NAN\;flow\;\times \;\%\;atom\\ & & \;excess\;of{\;}^{15}N\;in\;NAN\;effluent) \end{array}}{\left(\%\;atom\;excess\;of\;{}^{15}N\;in\;bacteria\;pellet\right)}, \end{array}$$

where

$\begin{array}{l} \%\;atom\;excess\;of{\;}^{15}N\;in\;NAN\;effluent\\ =\%\;ato{m\;}^{15}N\;in\;NAN\;effluent\;sample\\ -\%\;ato{m\;}^{15}N\;in\;background. \end{array}$

Additionally, the flow of dietary N and indicators of N metabolism by microorganisms were also determined according to Bach and Stern (1999) as follows:

$$\begin{array}{l} \begin{array}{ll} Dietary\;N\;flow\;\left(g/d\right)=\;\; & g\;of\;NAN\;in\;effluent\\ & -\;g\;of\;bacterial\;N\;\\ & in\;effluent, \end{array} \end{array}$$

$$\begin{array}{ll} Bacterial\;efficiency= & \;\;bacterial\;N\;flow\;(g)/OM\;truly\\ & \;digested\;\left(kg\right), \end{array}$$

$$\begin{array}{l} Efficiency\;of\;N\;use\;\left(ENU\right)=\left(\frac{g\;of\;bacterial\;N}{g\;of\;available\;N}\right)\times 100. \end{array}$$

Ruminal digestibility of OM, CP, and NDF was estimated according to Soder et al. (2013) by calculating the proportion of undigested nutrients in the digesta effluent and correcting for the contributions of artificial saliva and bacteria, as shown:

$$\begin{array}{l} \begin{array}{c} \%\;Nutrient\;digestibility\;\\ (DM\;basis)=100\;\times \end{array}\frac{\left[\begin{array}{c} g\;of\;nutrient\;intake\;\\ -(g\;of\;nutrient\;in\;effluent\\ -g\;of\;nutrient\;in\;saliva\\ -g\;of\;nutrient\;in\;bacteria) \end{array}\right]}{g\;of\;nutrient\;intake}\; \end{array}$$

### Statistical Analysis

Analysis of variance was performed with the MIXED procedure of SAS 9.4 (SAS Institute Inc., Cary, NC). Data were analyzed as a 4 × 4 Latin square design where the model included treatment as a fixed effect, and random effects of period and fermenter. Additionally, pH data were analyzed as repeated measures using the AR1 covariance matrix structure (based on lowest AIC criterion) and this model also included the fixed effects of time (expressed as hours after feed provision) and time × treatment. Multiple comparisons between treatments: 1) Control; 2) CO_3_; 3) OH; and 4) CO_3_/OH were evaluated with Tukey’s test. Moreover, the following orthogonal contrasts were evaluated: $\left[CaMg{\left(CO_{3}\right)}_{2}\right]=\;\left(CO_{3}+\;CO_{3}/OH\right)$ vs. Control to compare the effect of CaMg(CO_3_)_2_ as a source of Mg with the use of MgO plus a buffer; and $\left[CaMg{\left(OH\right)}_{4}\right]=\;\left(OH\;+\;CO_{3}/OH\right)$ vs. Control, representing the comparison between the use of CaMg(OH)_4_ as a source of Mg and the use of MgO plus a buffer. Significance was declared at *P* ≤ 0.05, while 0.05 < *P* < 0.10 was considered as trend.

## RESULTS AND DISCUSSION

### Ruminal pH and Concentration of Soluble Mg

Results on pH-related variables and concentration of soluble magnesium are shown in Table 2. Repeated measures analysis indicated that interaction treatment × time was not significant; therefore, pH kinetics data are not presented. Average pH of the ruminal fluid in the fermenters was similar across experimental treatments. Similarly, orthogonal contrasts show that the inclusion of either CaMg(CO_3_)_2_ or CaMg(OH)_4_ as sources of Mg in the diets, yields similar average pH compared to the Control treatment formulated with MgO and sodium sesquicarbonate.

**Table 2.** Effect of calcium magnesium carbonate and calcium magnesium hydroxide on pH and Mg solubility in a continuous culture system

Variable^*a*^	Treatment means^*b*^						*P*-value Orthogonal contrasts	
	Control	CO_3_	OH	CO_3_/OH	SEM	Trt^*c*^	[CaMg(CO_3_)_2_]^*d*^	[CaMg(OH)_4_]^*e*^
pH	6.28	6.18	6.20	6.27	0.06	0.20	0.28	0.37
pH-B6, %	12.5^b^	37.5^a^	20.0^b^	10.0^b^	6.4	<0.01	0.06	0.66
pH-AUC, pH•h	7.4^b^	22.3^a^	11.9^b^	6.0^b^	0.51	<0.01	0.05	0.65
Mg, mmol/L	1.42	1.30	1.35	1.27	0.17	0.73	0.31	0.39

^*a*^pH: daily average pH; pH-B6: % of time with a pH < 6.0; pH-AUC: area under the curve of pH for measurements below 6.0 (pH•h), Mg: concentration of soluble Mg in ruminal fluid effluent (mmol/L).

^*b*^Experimental treatments based on supplemental source of Mg: “Control” = 100% as MgO + sodium sesquicarbonate added as a buffer; “CO_3_” = 100% as CaMg(CO_3_)_2_; “OH” = 100% as CaMg(OH)_4_; “CO_3_/OH” = 50% as CaMg(CO_3_)_2_/50% as CaMg(OH)_4_. Means with different superscript within the same row are statistically different (*P* ≤ 0.05).

^*c*^Trt: effect of experimental treatment.

^*d*^[CaMg(CO_3_)_2_] = (CO_3_ + CO_3_/OH) vs. Control.

^*e*^[CaMg(OH)_4_] = (OH + CO_3_/OH) vs. Control.

On the other hand, pH-B6 was affected by treatment (*P* < 0.01) showing that CO_3_ treatment spent a greater percentage of total time below the pH 6.0 threshold when compared to the three other treatments. Consequently, orthogonal contrasts show that using CaMg(CO_3_)_2_ as a source of Mg tends to increase pH-B6 with respect to the Control ([CaMg(CO_3_)_2_]; *P* = 0.06), while pH-B6 with the inclusion of CaMg(OH)_4_ in the diet was similar to the Control. The area under the curve below pH 6.0 (pH-AUC) was also affected by treatment (*P* < 0.01), with the CO_3_ treatment having a greater pH-AUC than the other treatments, indicating more acidic conditions for CO_3_ in comparison to the other treatments. Similarly, orthogonal contrasts indicated that pH-AUC of the Control treatment with MgO and buffer was smaller than pH-AUC of diets formulated with CaMg(CO_3_)_2_ ([CaMg(CO_3_)_2_]; *P* = 0.05), but was similar to pH-AUC observed in diets with CaMg(OH)_4_ as a source of Mg.

Our results show a similar response on pH, pH-B6, and pH-AUC between the diets containing CaMg(OH)_4_ as a source of Mg and the Control diet formulated with MgO plus sodium sesquicarbonate. The lack of differences in this case indicates that inclusion of CaMg(OH)_4_ as a source of Mg in the diet, allowed for similar pH-related conditions than those observed in a Control diet with a commercial buffer and MgO. Conversely, under the conditions of this experiment, the inclusion of CaMg(CO_3_)_2_ as a source of Mg allowed for a more acidic fermentation than our positive Control diet.

Recently, Leno et al. (2017) reported greater DMI and milk fat yield during the first week of lactation in Holstein cows when CaMg(CO_3_)_2_ was supplemented during the peripartum compared to MgO supplemented cows. Ruminal fermentation was not evaluated in that trial, therefore it is not possible to know whether differences in DMI were related to ruminal pH or just different acceptance of MgO and CaMg(CO_3_)_2_ by the cows, indirectly impacting intake; although greater milk fat concentration may suggest possible changes in ruminal fermentation promoted by CaMg(CO_3_)_2_. Similarly, greater DMI has been already associated with the positive response in milk production observed in dairy cows supplemented with MgO (Bach et al., 2018), suggesting that the productive response to supplementation with inorganic sources of Mg can be, at least to some extent, driven by changes in intake.

The dual-flow continuous culture system allows to control the intake or supply of dry matter and nutrients. In the case of our experiment, the supply of nutrients was held constant across treatments; eliminating the effect of intake or preference that could be observed in other types of experiments, and consequently isolating the effect of treatments on ruminal fermentation; which in this case indicate that CaMg(OH)_4_ had a greater alkalinizing effect than CaMg(CO_3_)_2_.

We did not observe any effect of treatment on the concentration of soluble Mg in ruminal fluid in our experiment (Table 2), indicating similar solubilization among the sources we evaluated. Decreased ruminal absorption of Mg in sheep (Rahnema and Fontenot, 1983) and lower plasma Mg concentration in cows (Leno et al., 2017), have been reported when animals are fed CaMg(CO_3_)_2_ in comparison to MgO. However, none of these trials evaluated ruminal solubility of Mg; therefore, such differences in absorption or availability of Mg cannot be attributed to differences in ruminal solubility which has been estimated to explain most of the variation observed in the absorption of Mg among different sources (NRC, 2001; Jittakhot et al., 2004). Interestingly, the differences in reactivity between sources, that we observed in acetic acid solution, were not translated into differences in Mg solubility in ruminal fluid. Such discrepancy may be due to factors such as average pH, temperature, and fluctuation in pH, which differ between the acetic acid solution and the ruminal contents in the dual-flow culture system.

### Volatile Fatty Acids

Results of total VFA concentration and individual molar proportions are presented in Table 3. The total concentration of VFA and molar proportions of acetate and propionate were similar across treatments while there were no significant differences in the contrasts for these variables either. Butyrate molar proportion was greater in Control and CO_3_/OH treatments than in CO_3_ (Trt; *P* = 0.04); however, butyrate molar proportion in OH did not differ from CO_3_. Orthogonal contrasts indicated that the molar proportion of butyrate tended to be lower for diets supplemented with CaMg(CO_3_)_2_ compared to Control ([CaMg(CO_3_)_2_]; *P* = 0.08). Similarly, the molar proportion of caproate was lower in CO_3_ than in the other three treatments (Trt; *P* = 0.03).

**Table 3.** Effect of calcium magnesium carbonate and calcium magnesium hydroxide on total VFA concentration and individual molar proportions in pooled effluent in a continuous culture system

Item	Treatment means^*c*^						*P*-value Orthogonal contrasts	
	Control	CO_3_	OH	CO_3_/OH	SEM	Trt^*d*^	[CaMg(CO_3_)_2_]^*e*^	[CaMg(OH)_4_]^*f*^
Total VFA, mM	96.3	96.2	98.2	95.7	2.30	0.86	0.90	0.81
VFA, % of total VFA								
Acetate	54.1	54.7	53.7	54.5	1.28	0.57	0.45	0.99
Propionate	24.6	25.3	25.3	24.0	1.10	0.36	0.96	0.96
Butyrate	16.4^a^	15.0^b^	15.8^b^	16.3^a^	0.83	0.04	0.08	0.40
Isobutyrate	0.73	0.64	0.67	0.71	0.03	0.06	0.09	0.20
Isovalerate	1.51	1.77	1.62	1.72	0.18	0.07	0.01	0.08
Caproate	0.84^a^	0.66^b^	0.90^a^	0.92^a^	0.14	0.03	0.54	0.36
BCVFA^*a*^	2.23	2.42	2.28	2.43	0.19	0.34	0.09	0.27
A:P^*b*^	2.23	2.19	2.16	2.30	0.13	0.54	0.90	0.98

^*a*^BCVFA: branched-chain VFA.

^*b*^A:P: acetate to propionate ratio.

^*c*^Experimental treatments based on supplemental source of Mg: “Control” = 100% as MgO + sodium sesquicarbonate added as a buffer; “CO_3_” = 100% as CaMg(CO_3_)_2_; “OH” = 100% as CaMg(OH)_4_; “CO_3_/OH” = 50% as CaMg(CO_3_)_2_/50% as CaMg(OH)_4_. Means with different superscript within the same row are statistically different (*P* ≤ 0.05).

^*d*^Trt: effect of experimental treatment.

^*e*^[CaMg(CO_3_)_2_] = (CO_3_ + CO_3_/OH) vs. Control.

^*f*^[CaMg(OH)_4_] = (OH + CO_3_/OH) vs. Control.

Molar proportions of VFA resulting from amino acid degradation are also shown in Table 3. Both isobutyrate (*P* = 0.06) and isovalerate (*P* = 0.07) molar proportions tended to be affected by treatments. Inclusion of CaMg(CO_3_)_2_ as an Mg source in the diet promoted a greater molar proportion of isovalerate compared to the Control treatment ([CaMg(CO_3_)_2_]; *P* = 0.01), indicating greater deamination of leucine by ruminal microorganisms (Allison, 1978) when CaMg(CO_3_)_2_ is supplemented; which is reflected in a trend towards a greater BCVFA molar proportion in CaMg(CO_3_)_2_ treated diets compared to Control ([CaMg(CO_3_)_2_]; *P* = 0.09), and maybe indicative of either greater ruminal degradation of the branched-chain amino acid leucine or lesser utilization of isovalerate by ruminal microorganisms (Apajalahti, 2019).

Our results indicate that both the diets with CaMg(OH)_4_ as a supplemental source of Mg and the Control treatment with MgO and sodium sesquicarbonate may favor butyrate synthesis and a fermentation profile that could eventually allow for a greater energy supply to the ruminal epithelium (Kristensen and Harmon, 2004); similar to the in vivo response to bicarbonate supplementation on ruminal fermentation and production (Erdman, 1988; Staples and Lough). Moreover, although much less studied, caproate has been reported as a ketogenic VFA (Kristensen and Harmon, 2005) that also has a positive correlation with acetate synthesis in the rumen (Ertl et al., 2015).

In a summary of 82 experiments, Erdman (1988) found that cows consuming diets with corn silage as the source of forage, had lower propionate concentrations in ruminal fluid, and greater acetate concentration and acetate:propionate ratio when supplemented with sodium bicarbonate. Cruywagen et al. (2015) found an increase in ruminal acetate and butyrate, in response to supplementation with either bicarbonate or calcareous marine algae. More recently, Iwaniuk and Erdman (2015) evaluated the effect of the cation-anion difference of the diet on performance of dairy cows supplemented with buffers; their meta-analysis showed a positive association between dietary cation–anion difference and both acetate and butyrate molar percentages. The similar response on butyrate molar proportion observed in our experiment between diets with CaMg(OH)_4_ inclusion and Control diet in addition to the response on ruminal pH is consistent with some of these reports in cows supplemented with buffers which seem to stimulate the ruminal synthesis of lipogenic VFA.

### Flows of N and N Metabolism

The effects of CaMg(CO_3_)_2_ and CaMg(OH)_4_ on the concentration of NH_3_-N were analyzed in samples of digesta effluent and used for estimation of N flows which are shown in Table 4. There were no effects of treatment on NH_3_-N concentration, total N flow, NH_3_-N flow, or NAN flow. None of these variables were altered by CaMg(CO_3_)_2_ or CaMg(OH)_4_ inclusion when compared to the Control diet according to the orthogonal contrasts analysis. Nevertheless, within the fraction corresponding to NAN flow, the flow of bacterial N tended to be influenced by treatment (Trt; *P* = 0.06) and also was lower when diets containing CaMg(CO_3_)_2_ were compared to the positive Control containing MgO plus buffer ([CaMg(CO_3_)_2_]; *P* = 0.04). Moreover, inclusion of CaMg(CO_3_)_2_ in the diet also tended to decrease the efficiency of utilization of available N ([CaMg(CO_3_)_2_]; *P* = 0.06) and digestible OM ([CaMg(CO_3_)_2_]; *P* = 0.08) for synthesis of bacterial N.

**Table 4.** Effect of calcium magnesium carbonate and calcium magnesium hydroxide on N metabolism in a continuous culture system

Variable	Treatment means^*g*^						*P*-value Orthogonal contrasts	
	Control	CO_3_	OH	CO_3_/OH	SEM	Trt^*h*^	[CaMg(CO_3_)_2_]^*i*^	[CaMg(OH)_4_]^*j*^
NH_3_-N, mg/dL	17.6	16.0	17.2	16.5	1.06	0.44	0.17	0.41
N flows, g/d								
Total N	3.06	2.87	3.14	2.99	0.13	0.49	0.43	0.95
NH_3_-N^*a*^	0.71	0.65	0.69	0.67	0.04	0.49	0.18	0.43
NAN^*b*^	2.35	2.22	2.45	2.33	0.12	0.59	0.62	0.77
Bacterial N^*c*^	1.31	1.14	1.34	1.14	0.06	0.06	0.04	0.31
Dietary N^*d*^	1.03	1.08	1.11	1.19	0.08	0.53	0.29	0.23
ENU^*e*^	47.4	41.0	48.3	40.7	3.01	0.12	0.06	0.35
Bacterial efficiency^*f*^	20.4	17.9	20.8	18.1	1.14	0.14	0.08	0.44

^*a*^NH_3_-N (g/d) = mg/dL of effluent NH_3_-N × (g of total effluent flow/100).

^*b*^NAN = nonammonia N flow (g/d) = g of effluent N − g of effluent NH_3_-N.

^*c*^Bacterial-N flow (g/d) = (NAN flow × atom percentage excess of ^15^N of effluent)/(atom percentage excess of ^15^N of bacteria), according to Calsamiglia et al. (1996).

^*d*^Dietary N flow (g/d) = g of effluent NAN − g of effluent bacterial N.

^*e*^ENU = efficiency of N use = (g of bacterial N/g of available N) × 100 (Bach and Stern, 1999).

^*f*^Bacterial efficiency = g of bacterial N flow/kg of OM truly digested, according to Calsamiglia et al. (1996).

^*g*^Experimental treatments based on supplemental source of Mg: “Control” = 100% as MgO + sodium sesquicarbonate added as a buffer; “CO_3_” = 100% as CaMg(CO_3_)_2_; “OH” = 100% as CaMg(OH)_4_; “CO_3_/OH” = 50% as CaMg(CO_3_)_2_/50% as CaMg(OH)_4_. Means with different superscript within the same row are statistically different (*P* ≤ 0.05).

^*h*^Trt: effect of experimental treatment.

^*i*^[CaMg(CO_3_)_2_] = (CO_3_ + CO_3_/OH) vs. Control.

^*j*^[CaMg(OH)_4_] = (OH + CO_3_/OH) vs. Control.

As discussed before, our results on isovalerate and BCVFA shown in Table 3 indicate either a greater ruminal degradation of leucine or reduced utilization of isovalerate by ruminal microorganisms, allowing for a greater molar proportion of this VFA when CaMg(CO_3_)_2_ was fed in comparison to Control diet. Based on the results of N metabolism, a possible explanation is that isovalerate accumulates in the ruminal fluid as a consequence of reduced uptake by ruminal bacteria due to a decreased microbial synthesis when CaMg(CO_3_)_2_ is used as the supplemental source of Mg in the diet. Ruminal cellulolytic bacteria require BCVFA as growth factors due to their inability to directly incorporate preformed amino acids which makes them dependent on BCVFA and NH_3_ to perform bacterial protein synthesis (Dehority et al., 1967). Then, in this case, a trend towards a greater molar proportion of BCVFA observed in diets containing CaMg(CO_3_)_2_ compared to Control, may be indicative of decreased growth rate of cellulolytic bacteria; however bacterial populations analysis would be required to evaluate this possibility.

Decreased bacterial N flow influenced by lower ruminal pH in a dual-flow continuous culture was reported by Calsamiglia et al. (2008) who estimated that 39% of the variation in bacterial N flow was explained by pH. Although they did not evaluate bacterial populations, a possible explanation for limited bacterial growth at lower pH is the greater maintenance requirements and consequently lower growth efficiency of nonstructural carbohydrates-degrading bacteria compared to cellulolytic bacteria (Russell et al., 1992).

### Nutrient Digestibility

As shown in Table 5, digestibility of OM, CP, and NDF was similar among treatments. Additionally, the inclusion of either CaMg(CO_3_)_2_ or CaMg(OH)_4_ in the diet allowed for similar values of nutrient digestibility than the Control diet, as indicated by orthogonal contrasts. In two reviews summarizing 82 and 41 experiments, respectively, Erdman (1988) and Staples and Lough (1989) reported greater digestibility of DM and NDF or ADF in cows fed corn-silage based diets when supplemented with 1% sodium bicarbonate or sesquicarbonate at 0.75% to 1% of diet DM. Moreover, supplementing MgO at 0.4% to 0.8% of DM increased fat corrected milk yield compared to non-supplemented cows (Staples and Lough, 1989). However, when cows were fed a different source of forage, such as alfalfa hay, or another source of fiber was included in the diet, such as cottonseed hulls, there was no response to buffers supplementation, showing that effectivity of buffers depends on the type of diet and that corn silage based diets with a rather high concentration of starch (>30%) are the ones that show a better response (Erdman, 1988). Our experimental diets met those criteria offering the right conditions for buffer effectiveness, which means that adequate substrate was provided for the synthesis of organic acids from microbial fermentation of starch and sugars, and within that context, the inclusion of CaMg(CO_3_)_2_ and CaMg(OH)_4_ had a similar effect on nutrient digestibility, and such effect was also comparable to that one of the Control diets with MgO plus buffer.

**Table 5.** Effect of calcium magnesium carbonate and calcium magnesium hydroxide on nutrient digestibility in a continuous culture system

Digestibility, %^*a*^	Treatment means^*b*^						*P*-value Orthogonal contrasts	
	Control	CO_3_	OH	CO_3_/OH	SEM	Trt^*c*^	[CaMg(CO_3_)_2_]^*d*^	[CaMg(OH)_4_]^*e*^
OM	64.0	63.6	64.1	62.9	1.56	0.83	0.56	0.70
CP	62.9	61.3	60.2	57.1	2.71	0.52	0.29	0.23
NDF	57.6	57.9	55.7	59.1	2.68	0.84	0.81	0.94

^*a*^True digestibility of organic matter (OM), crude protein (CP) and neutral detergent fiber (NDF).

^*b*^Experimental treatments based on supplemental source of Mg: “Control” = 100% as MgO + sodium sesquicarbonate added as a buffer; “CO_3_” = 100% as CaMg(CO_3_)_2_; “OH” = 100% as CaMg(OH)_4_; “CO_3_/OH” = 50% as CaMg(CO_3_)_2_/50% as CaMg(OH)_4_. Means with different superscript within the same row are statistically different (*P* ≤ 0.05).

^*c*^Trt: effect of experimental treatment.

^*d*^[CaMg(CO_3_)_2_] = (CO_3_ + CO_3_/OH) vs. Control.

^*e*^[CaMg(OH)_4_] = (OH + CO_3_/OH) vs. Control.

Although it is still unclear the mechanism on how ruminal buffers and alkalizers alter ruminal digestibility (Iwaniuk and Erdman, 2015), it has been shown that reducing ruminal acidity by the inclusion of these products in the diets of dairy cows, not only can increase the digestibility of NDF, but also may modify lipid biohydrogenation pattern in the rumen by increasing synthesis of *cis*-9, *trans*-11 conjugated linoleic acid (Jenkins et al., 2014); which explains part of the response observed in milk fat synthesis of animals supplemented with ruminal pH modifiers. We did not evaluate lipid biohydrogenation in our experiment; therefore, we can only discard a difference in NDF digestibility between our treatments.

Moreover, all the diets in our experiment were formulated to the same concentration of Mg based on the NRC’s recommendations (2001). Therefore, our results rule out the confounding effect of a different concentration of Mg among treatments and focus on the effect of source of supplemental Mg, and the inclusion of buffer in the case of the Control treatment; indicating that although it had an impact on the pattern of VFA synthesis, it may have not been such a big change as to modify nutrient digestibility.

Therefore, under the conditions of this experiment, the inclusion of CaMg(OH)_4_ in diets as a source of Mg, either alone or combined with CaMg(CO_3_)_2_, allowed for a less acidic ruminal fermentation pattern than a diet formulated with CaMg(CO_3_)_2_ as the only supplemental source of Mg. Moreover, using CaMg(OH)_4_, either alone or combined with CaMg(CO_3_)_2_, allowed similar microbial fermentation variables than a positive Control diet formulated with MgO plus sodium sesquicarbonate as a buffer. Further research would be needed to evaluate in vivo response to supplementation with these sources of Mg and also their availability for ruminants.

## References

[CIT0001] Allison M. J 1978 Production of branched-chain volatile fatty acids by certain anaerobic bacteria. Appl. Environ. Microbiol. 35:872–877. doi:10.1128/AEM.35.5.872-877.1978 566082 PMC242945

[CIT0002] Ammerman C. B., Chicco C. F., Loggins P. E., and Arrington L. R.. 1972 Availability of different inorganic salts of magnesium to sheep. J. Anim. Sci. 34:122–126. doi:10.2527/jas1972.341122x 5059168

[CIT0003] AOAC. 1990 Ofﬁcial methods of analysis. 13th ed. Washington, DC: Assoc. Off. Anal. Chem.

[CIT0004] AOAC. 2000 Ofﬁcial methods of analysis. 17th ed. Washington, DC: Assoc. Off. Anal. Chem.

[CIT0005] Apajalahti J., Vienola K., Raatikainen K., Holder V., and Moran C. A.. 2019 Conversion of branched-chain amino acids to corresponding isoacids. An in vitro tool for estimating ruminal protein degradability. Front. Vet. Sci. 6:311. doi:10.3389/fvets.2019.00311 31620454 PMC6759480

[CIT0006] Bach A., Guasch I., Elcoso G., Duclos J., and Khelil-Arfa H.. 2018 Modulation of rumen pH by sodium bicarbonate and a blend of different sources of magnesium oxide in lactating dairy cows submitted to a concentrated challenge. J. Dairy Sci. 101:9777–9788. doi:10.3168/jds.2017-14353 30172393

[CIT0007] Bach A. and Stern M. D.. 1999 Effects of different levels of methionine and ruminally undegradable protein on the amino acid profile of effluent from continuous culture fermenters. J. Anim. Sci. 77:3377–3384. doi:10.2527/1999.77123377x 10641887

[CIT0008] Brandao V. L. N., and Faciola A. P.. 2019 Unveiling the relationships between diet composition and fermentation parameters response in dual-flow continuous culture system: a meta-analytical approach. Transl. Anim. Sci. 3:1064–1075. doi:10.1093/tas/txz019 32704870 PMC7200414

[CIT0009] Brandao V. L. N., Marcondes M. I., and Faciola A. P.. 2020 Comparison of microbial fermentation data from dual-flow continuous culture system and omasal sampling technique: a meta-analytical approach. J. Dairy Sci. 103:2347–2362. doi:10.3168/jds.2019-17107 31954580

[CIT0010] Broderick G. A., and Kang J. H.. 1980 Automated simultaneous determination of ammonia and total amino acids in ruminal fluid and in vitro media. J. Dairy Sci. 63:64–75. doi:10.3168/jds.S0022-0302(80)82888-8 7372898

[CIT0011] Calsamiglia S., Cardozo P. W., Ferret A., and Bach A.. 2008 Changes in rumen microbial fermentation are due to a combined effect of type of diet and pH. J. Anim. Sci. 86:702–711. doi:10.2527/jas.2007-0146 18073289

[CIT0012] Calsamiglia S., Stern M. D., and Firkins J. L.. 1996 Comparison of nitrogen-15 and purines as microbial markers in continuous culture. J. Anim. Sci. 74:1375–1381. doi:10.2527/1996.7461375x 8791211

[CIT0013] Cardoso C., Sears W., LeBlanc S. J., and Drackley J. K.. 2011 Technical note: comparison of 3 methods for analyzing areas under the curve for glucose and nonesterified fatty acids concentrations following epinephrine challenge in dairy cows. J. Dairy Sci. 94:6111–6115. doi:10.3168/jds.2011-4627 22118098

[CIT0014] Cruywagen C. W., Taylor S., Beya M. M., and Calitz T.. 2015 The effect of buffering dairy cow diets with limestone, calcareous marine algae, or sodium bicarbonate on ruminal pH profiles, production responses, and rumen fermentation. J. Dairy Sci. 98:5506–5514. doi:10.3168/jds.2014-8875 26026755

[CIT0015] Dehority B. A., Scott H. W., and Kowaluk P.. 1967 Volatile fatty acid requirements of cellulolytic rumen bacteria. J. Bacteriol. 94:537–543. doi:10.1128/JB.94.3.537-543.1967 6068143 PMC251919

[CIT0016] Erdman R. A 1988 Dietary buffering requirements of the lactating dairy cow: a review. J. Dairy Sci. 71:3246–3266. doi:10.3168/jds.S0022-0302(88)79930-0

[CIT0017] Erdman R. A., Hemken R. W., and Bull L. S.. 1982 Dietary sodium bicarbonate and magnesium oxide for early postpartum lactating dairy cows: effects of production, acid-based metabolism, and digestion. J. Dairy Sci. 65:712–731. doi:10.3168/jds.S0022-0302(82)82259-5 6286736

[CIT0018] Ertl P., Knaus W., Metzler-Zebeli B. U., Klevenhusen F., Khiaosa-Ard R., and Zebeli Q.. 2015 Substitution of common concentrates with by-products modulated ruminal fermentation, nutrient degradation, and microbial community composition in vitro. J. Dairy Sci. 98:4762–4771. doi:10.3168/jds.2014-9063 25981072

[CIT0019] Goff J. P 2018 Invited review: Mineral absorption mechanisms, mineral interactions that affect acid–base and antioxidant status, and diet considerations to improve mineral status. J. Dairy Sci. 101:2763–2813. doi:10.3168/jds.2017-13112 29397180

[CIT0020] Hoover W. H., Crooker B. A., and Sniffen C. J.. 1976 Effects of differential solid-liquid removal rates on protozoa numbers in continuous cultures of rumen contents. J. Anim. Sci. 43:528–534. doi:10.2527/jas1976.432528x

[CIT0021] Iwaniuk M. E., and Erdman R. A.. 2015 Intake, milk production, ruminal, and feed efficiency responses to dietary cation-anion difference by lactating dairy cows. J. Dairy Sci. 98:8973–8985. doi:10.3168/jds.2015-9949 26409960

[CIT0022] Jenkins T. C., Bridges W. C. Jr., Harrison J. H., and Young K. M.. 2014 Addition of potassium carbonate to continuous cultures of mixed ruminal bacteria shifts volatile fatty acids and daily production of biohydrogenation intermediates. J. Dairy Sci. 97:975–984. doi:10.3168/jds.2013-7164 24359822

[CIT0023] Jittakhot S., Schonewille J. T., Wouterse H., Yuangklang C., and Beynen A. C.. 2004 Apparent magnesium absorption in dry cows fed at 3 levels of potassium and 2 levels of magnesium intake. J. Dairy Sci. 87:379–385. doi:10.3168/jds.S0022-0302(04)73177-X 14762081

[CIT0024] Kristensen N. B., and Harmon D. L.. 2004 Splanchnic metabolism of volatile fatty acids absorbed from the washed reticulorumen of steers. J. Anim. Sci. 82:2033–2042. doi:10.2527/2004.8272033x 15309950

[CIT0025] Kristensen N. B., and Harmon D. L.. 2005 Effects of adding valerate, caproate, and heptanoate to ruminal buffers on splanchnic metabolism in steers under washed-rumen conditions. J. Anim. Sci. 83:1899–1907. doi:10.2527/2005.8381899x 16024710

[CIT0026] Krizsan S. J., Ahvenjärvi S., Volden H., and Broderick G. A.. 2010 Estimation of rumen outflow in dairy cows fed grass silage-based diets by use of reticular sampling as an alternative to sampling from the omasal canal. J. Dairy Sci. 93:1138–1147. doi:10.3168/jds.2009-2661 20172235

[CIT0027] Leno B. M., LaCount S. E., Ryan C. M., Briggs D., Crombie M., and Overton T. R.. 2017 The effect of source of supplemental dietary calcium and magnesium in the peripartum period, and level of dietary magnesium postpartum, on mineral status, performance, and energy metabolites in multiparous Holstein cows. J. Dairy Sci. 100:7183–7197. doi:10.3168/jds.2017-12773 28711248

[CIT0028] National Research Council (NRC). 2001 Nutrient requirements of dairy cattle. 7th revised ed Washington, DC: The National Academies Press.

[CIT0029] Rahnema S. H., and Fontenot J. P.. 1983 Effect of supplemented magnesium from magnesium oxide or dolomitic limestone upon digestion and absorption of minerals in sheep. J. Anim. Sci. 57:1545–1552. doi:10.2527/jas1983.5761545x 6674291

[CIT0030] Ruiz-Moreno M., Binversie E., Fessenden S. W., and Stern M. D.. 2015 Mitigation of in vitro hydrogen sulfide production using bismuth subsalicylate with and without monensin in beef feedlot diets. J. Anim. Sci. 93:5346–5354. doi:10.2527/jas.2015-9392 26641054

[CIT0031] Russell J. B., O’Connor J. D., Fox D. G., Van Soest P. J., and Sniffen C. J.. 1992 A net carbohydrate and protein system for evaluating cattle diets: I. Ruminal fermentation. J. Anim. Sci. 70:3551–3561. doi:10.2527/1992.70113551x 1459918

[CIT0032] Schonewille J. T., and Beynen A. C.. 2005 Reviews on the mineral provision in ruminants (III): magnesium metabolism and requirements in ruminants. CVB documentation report N° 35. Utrecht, The Netherlands: Department of Nutrition, Faculty of Veterinary Medicine Utrecht University.

[CIT0033] Soder K. J., Brito A. F., and Rubano M. D.. 2013 Effect of supplementing orchardgrass herbage with a total mixed ration or flaxseed on fermentation profile and bacterial protein synthesis in continuous culture. J. Dairy Sci. 96:3228–3237. doi:10.3168/jds.2012-6307 23522677

[CIT0034] Staples C. R., and Lough D. S.. 1989 Efficacy of supplemental dietary neutralizing agents for lactating dairy cows. A review. Anim. Feed Sci. Tech. 23:277–303. doi:10.1016/0377-8401(89)90050-3

[CIT0035] Van Soest P. J., Robertson J. B., and Lewis B. A.. 1991 Methods for dietary fiber, neutral detergent fiber, and nonstarch polysaccharides in relation to animal nutrition. J. Dairy Sci. 74:3583–3597. doi:10.3168/jds.S0022-0302(91)78551-2 1660498

[CIT0036] Weller R. A., and Pilgrim A. F.. 1974 Passage of protozoa and volatile fatty acids from the rumen of the sheep and from a continuous in vitro fermentation system. Br. J. Nutr. 32:341–351. doi:10.1079/bjn19740087 4213614

